# Patient-level explainable machine learning to predict major adverse cardiovascular events from SPECT MPI and CCTA imaging

**DOI:** 10.1371/journal.pone.0291451

**Published:** 2023-11-15

**Authors:** Fares Alahdab, Radwa El Shawi, Ahmed Ibrahim Ahmed, Yushui Han, Mouaz Al-Mallah

**Affiliations:** 1 Houston Methodist DeBakey Heart & Vascular Center, Houston, TX, United States of America; 2 Institute of Computer Science, University of Tartu, Tartu, Estonia; Institute for Basic Science, REPUBLIC OF KOREA

## Abstract

**Background:**

Machine learning (ML) has shown promise in improving the risk prediction in non-invasive cardiovascular imaging, including SPECT MPI and coronary CT angiography. However, most algorithms used remain black boxes to clinicians in how they compute their predictions. Furthermore, objective consideration of the multitude of available clinical data, along with the visual and quantitative assessments from CCTA and SPECT, are critical for optimal patient risk stratification. We aim to provide an explainable ML approach to predict MACE using clinical, CCTA, and SPECT data.

**Methods:**

Consecutive patients who underwent clinically indicated CCTA and SPECT myocardial imaging for suspected CAD were included and followed up for MACEs. A MACE was defined as a composite outcome that included all-cause mortality, myocardial infarction, or late revascularization. We employed an Automated Machine Learning (AutoML) approach to predict MACE using clinical, CCTA, and SPECT data. Various mainstream models with different sets of hyperparameters have been explored, and critical predictors of risk are obtained using explainable techniques on the global and patient levels. Ten-fold cross-validation was used in training and evaluating the AutoML model.

**Results:**

A total of 956 patients were included (mean age 61.1 ±14.2 years, 54% men, 89% hypertension, 81% diabetes, 84% dyslipidemia). Obstructive CAD on CCTA and ischemia on SPECT were observed in 14% of patients, and 11% experienced MACE. ML prediction’s sensitivity, specificity, and accuracy in predicting a MACE were 69.61%, 99.77%, and 96.54%, respectively. The top 10 global predictive features included 8 CCTA attributes (segment involvement score, number of vessels with severe plaque ≥70, ≥50% stenosis in the left marginal coronary artery, calcified plaque, ≥50% stenosis in the left circumflex coronary artery, plaque type in the left marginal coronary artery, stenosis degree in the second obtuse marginal of the left circumflex artery, and stenosis category in the marginals of the left circumflex artery) and 2 clinical features (past medical history of MI or left bundle branch block, being an ever smoker).

**Conclusion:**

ML can accurately predict risk of developing a MACE in patients suspected of CAD undergoing SPECT MPI and CCTA. ML feature-ranking can also show, at a sample- as well as at a patient-level, which features are key in making such a prediction.

## Introduction

In recent years, there has been a surge in the use of machine learning (ML) techniques in cardiovascular imaging. As the number of imaging modalities for evaluating patients with potential coronary artery disease (CAD) increases and the technology continues to improve, there is an abundance of data available to consider when making clinical judgments. However, the large number of variables and growing volume of imaging data can make it challenging to accurately assess patients. Artificial intelligence (AI) and ML can assist in this process by providing helpful prompts based on a wide range of clinical and imaging variables [1]. Indeed, ML algorithms have been shown to be valuable tools in patient risk stratification and diagnostic assessments [2,3]. Coronary computed tomography angiography (CCTA) is a non-invasive diagnostic procedure used to assess coronary arteries for CAD. It has a high negative predictive value, allowing a negative CCTA result effectively rules out significant CAD [4,5]. Another important non-invasive diagnostic test is single photon emission computed tomography (SPECT) which mainly assesses the functional significance of coronary stenosis and guides management. Assessment of plaque and perfusion burden using CCTA and SPECT add incremental prognostic value in patients suspected of CAD [6–8].

The prevalent approach to clinical prediction typically involves selecting potentially relevant variables by experts, followed by regression/classification analysis. Recent advancements in ML render this classical approach restrictive (uses only one model type), inefficient (requires manual tunning for hyperparameters) and potentially biased (predictor pre-selection). AutoML aims to alleviate the computational cost and human expertise required to develop well-performing ML pipelines [9,10]. Despite advancements in ML-based prediction models in the healthcare, one major obstacle to the adoption of these models is that many of them are considered “black boxes”, which refer to the lack of interpretability [11]. There have been calls for more research on how these models operate [12–15]. The inability to interpret predictive models can erode trust in them, particularly in cardiovascular medicine where decisions can have serious consequences. In medicine, black box models will have a significant role and, in many cases, are not too different from other areas where we lack complete biological or clinical understanding [16]. However, just as it is beneficial to understand the mechanisms behind diseases and therapies, it can be helpful to have greater understanding of how ML models arrive at their conclusions [17]. There has been a surge in research on explainable ML in an effort to address this issue [18]. Various methods for exploring the reasoning behind AI predictions have been developed [19,20]. One effective method is to build a secondary, more transparent model, such as a decision tree or random forest, through which the input variables can be traced to the final predictor variables, and the features most important for prediction can be identified [21–23].

This study aimed to develop an ML model using AutoML technique to predict major adverse cardiac events (MACE) via various data parameters obtained from clinical, CCTA, and SPECT evaluations of patients suspected of CAD. To gain trust in the prediction of the developed model, we explain the predictions on both the global and patient levels.

## Methods

### Study population

This retrospective study included consecutive patients who underwent clinically indicated CCTA and SPECT within 180 days of each other at the Houston Methodist DeBakey Heart and Vascular Center in Houston, Texas. The study period was from January 1, 2010, to June 22, 2020, and included 956 eligible patients without known CAD. Patients who had undergone revascularization or experienced a non-fatal myocardial infarction between the two diagnostic assessments, as well as those with congenital abnormalities of the coronary arteries or severe valvular disease, were excluded. The protocol for this study was approved by the Institutional Review Board at the Houston Methodist Academic Institute, and informed consent was waived.

### CCTA

CCTA scans were obtained using 3rd generation SOMATOM FORCE Scanner (Siemens, Forchheim, Germany) after 2016 (n = 530) and Phillips 64 slice CT (Philips Healthcare, Amsterdam, Netherlands) before 2016 (n = 426). Image acquisition was performed in accordance with the Society of Cardiovascular Computed Tomography (SCCT) guidelines [24]. Patients with a heart rate of 65 beats per minute or higher were given intravenous metoprolol, and 0.4 mg sublingual nitroglycerin was given to all patients immediately before image acquisition. During image acquisition, 60–100 CCs of contrast were injected, followed by saline flush. Axial scans were obtained with prospective electrocardiographic gating. The image acquisition included the coronary arteries, left ventricle, and proximal ascending aorta.

The images were evaluated using a 3D workstation, with various post-processing techniques such as axial, multiplanar reformat, maximum intensity projection, and cross-sectional analysis. Type and location of lesion were visually evaluated using an 18-segment model according to SCCT guidelines [24]. Atherosclerosis in each segment was defined as tissue structures larger than 1 mm2 within or adjacent to the coronary artery lumen that could be distinguished from pericardial tissue, epicardial fat, or the vessel lumen.

The percent coronary stenosis was determined by comparing the luminal diameter of the obstructed segment to the luminal diameter of the most normal-looking site and classified as none (0%), mild (1%–49%), moderate (50%–69%), or severe (≥70%) based on the degree of narrowing of the luminal diameter. Anatomically obstructive CAD by CCTA was defined as at least 50% stenosis in the left main artery and at least 70% stenosis in the proximal, mid, and distal branches of the left anterior descending, left circumflex, and right coronary artery, but not including side branches. Findings were reported using SCCT Coronary Artery Disease Reporting & Data System (CAD-RADS) [25]. Segment involvement score was used to quantify burden of disease using CCTA. Using an 18-segment coronary artery model, the presence of plaque in each segment was scored as 0 or 1, regardless of the degree of stenosis. The sum of all involved segments was calculated for each patient. Plaques were classified as non-calcified (NC SIS) or calcified/partially calcified (C/PC SIS) based on a Hounsfield unit threshold of <130. Calcified/partially calcified plaques were further divided into calcified (C SIS) and partially calcified (PC SIS) based on the uniformity of calcification.

### SPECT MPI

SPECT MPI scans were obtained using either an INTIVO scanner (Siemens, Forchheim, Germany) or a Phillips Brightview scanner (Philips Healthcare, Amsterdam, Netherlands). Image acquisition was performed in accordance with the American Society of Nuclear Cardiology guidelines [26]. Gated SPECT stress and stress-rest were performed using either a 1- or 2-day protocol as appropriate, with Regadenoson as the stressing agent. Gated end systolic and diastolic left ventricular volumes were used to calculate the ejection fraction. Perfusion was graded on a 5-point scale in all segments, and summed stress, rest, and difference scores were evaluated [27]. Scar was defined as a summed rest score >0, ischemia as a summed difference score >0, and significant ischemia as a summed difference score ≥7. All studies were interpreted by experienced imaging cardiologists who have at least 10 years of experience.

### Follow-up and outcome data

Patients’ clinical history, comorbidities, medications used, laboratory testing, and previous diagnostic modalities were captured. Medical records of enrolled patients were checked to record updates in relevant clinical and laboratory endpoints. The primary outcome was major adverse cardiovascular events (MACE), which is a composite of all-cause death, myocardial infarction (MI), and late revascularization (PCI or CABG) occurring more than 90 days after the earliest imaging date. MI was defined according to the 4th universal definition of myocardial infarction [28]. All patients were followed from the date of their first imaging study. Patients were censored at either the occurrence of outcomes or the last known date of contact with the healthcare system as recorded in their medical records.

### Machine learning methodology

#### Feature selection

The dataset of this study included 42 clinical variables,12 SPECT variables, and 83 CCTA variables. A full list of variables is outlined in S1 Table in S1 File. The main goal of feature selection is to improve the performance of a predictive model and reduce the computational cost of modelling. In this work, we used a feature selection technique, ANOVA F-test, to reduce the high dimensionality of the feature space before the modelling phase [29]. ANOVA is a set of parametric statistical models and their estimation procedures determining whether the means of two or more samples come from the same distribution. F-statistic is a set of statistical tests used to calculate the ratio between and within group variance. ANOVA F-test is a univariate statistical test where each feature is compared to the target feature to check the statistical relationship between them.

#### AutoML method

Our framework employs a state-of-the-art AutoML framework, AutoSklearn [30], for model selection and hyperparameter optimization to establish the predictive model of MACE. AutoSKlearn is implemented on top of Scikit-Learn [31] (using 15 classifiers, 14 feature preprocessing methods, and 4 data preprocessing methods, giving rise to a structured hypothesis space with 110 hyperparameters) and uses SMAC for algorithm selection and hyperparameter tuning. AutoSKlearn combines the best-performing models into a single ensemble, using ensemble selection methodology [32], to improve the performance and robustness of the output model. Ensemble selection is a greedy approach that starts with an empty ensemble and iteratively adds models to the ensemble to maximize the validation performance. Models were optimized and evaluated by area under receiver operating characteristic (ROC) curves (AUC). The final ensemble model obtained from AutoSKlearn was trained and evaluated using a 10-fold cross-validation approach (8 folds were used for training, 1 for validation, and 1 testing, repeated in 10 iterations).

### ML model explainability

To gain trust from clinical users and regulatory bodies in the predictions made by the ensemble model obtained from AutoSKlearn, we explain the model’s behavior on both global and patient levels. For global explanation, we utilized the permutation feature importance technique that measures the decrease in the prediction performance of the model after we permuted the feature’s values, which breaks the relationship between the feature and the true outcome [33]. Thus, the drop in the model performance indicates how much the model depends on the feature. The more significant the drop in performance, the more important the feature is to the model and vice versa. For patient-level (local) explanation, we utilized Local interpretable model-agnostic explanations (LIME) for explaining the predictions of any classifier in an interpretable and faithful manner, by learning an interpretable model locally around the prediction [34]. LIME highlights the main features that contribute toward and against the prediction of a specific patient.

### Statistical analysis

Variables that could take on any value within a certain continuous range were shown as a mean with a standard deviation, while variables that fell into specific categories were shown as a proportion with a percentage. These were compared using either a student’s t-test or a chi-square test, depending on the type of variable. Accuracy measures, including sensitivity, specificity, positive and negative predictive values, as well as likelihood ratios, were calculated and reported along with their respective 95% confidence intervals. All analyses were done using Stata 16.0 (StataCorp, College Station, Texas) and a two-tailed p-value of 0.05 was considered statistically significant.

## Results

### Baseline characteristics

The study included 956 patients (mean age 61.1 ±14.2 years, 54% men, 89% hypertension, 81% diabetes, 84% dyslipidemia). The median interval between SPECT and CCTA was 15 days (interquartile range: 2–101). 60% of the patients had imaging within 30 days. In the cohort, over three-fourths of the patients underwent CCTA either on the same day as SPECT or after SPECT (200 CT then SPECT vs 756 SPECT then CT).

Baseline characteristics of the study population are summarized in Table 1. Most patients (56%) were experiencing symptoms such as chest pain or shortness of breath, and 80% were taking aspirin or clopidogrel. Table 2 shows the CCTA and SPECT findings, while Table 3 shows the clinical outcomes in this cohort.

**Table 1:** Demographics and baseline characteristics of the enrolled participants.

Sociodemographic characteristics	Total	CCTA obstructive stenosis			SPECT—ischemia		
		No	Yes	*p*	No	Yes	*p*
	N = 956	N = 818	N = 138		N = 818	N = 138	
Age–Mean (SD)	61.14 (14.20)	59.97 (14.21)	68.04 (12.05)	<0.001	61.02 (14.26)	61.84 (13.87)	0.53
Sex–Female N (%)	439 (46%)	386 (47%)	53 (38%)	0.055	385 (47%)	54 (39%)	0.084
**Comorbidities**							
Hypertension–N (%)	848 (89%)	719 (88%)	129 (93%)	0.055	720 (88%)	128 (93%)	0.10
Diabetes–N (%)	778 (81%)	659 (81%)	119 (86%)	0.11	657 (80%)	121 (88%)	0.040
Dyslipidemia–N (%)	806 (84%)	674 (82%)	132 (96%)	<0.001	680 (83%)	126 (91%)	0.015
Heart failure–N (%)	107 (11%)	86 (11%)	21 (15%)	0.10	85 (10%)	22 (16%)	0.056
Ever smoker–N (%)	317 (33%)	257 (31%)	60 (43%)	0.005	263 (32%)	54 (39%)	0.11
**Symptoms**							
Chest pain or shortness of breath–N (%)	537 (56%)	474 (58%)	63 (46%)	0.007	472 (58%)	65 (47%)	0.020
**Medication**							
Aspirin/Clopidogrel–N (%)	768 (80%)	645 (79%)	123 (89%)	0.005	647 (79%)	121 (88%)	0.019
Statin–N (%)	706 (74%)	582 (71%)	124 (90%)	<0.001	589 (72%)	117 (85%)	0.002
ACE/ARB–N (%)	625 (65%)	525 (64%)	100 (72%)	0.059	527 (64%)	98 (71%)	0.13
Beta blockers–N (%)	770 (81%)	654 (80%)	116 (84%)	0.26	645 (79%)	125 (91%)	0.001
Calcium channel blockers–N (%)	431 (45%)	359 (44%)	72 (52%)	0.070	369 (45%)	62 (45%)	0.97

**Table 2:** Findings on the coronary computed tomography angiography (CCTA) and single-photon emission computed tomography (SPECT) imaging.

	Total N = 956		CCTA obstructive stenosis			SPECT—ischemia		
			No	Yes	*P value*	No	Yes	*P value*
			N = 818	N = 138		N = 818	N = 138	
**CCTA stenosis**								
CCTA CAD-RAD–N (%)					<0.001			<0.001
CAD RAD 0	288 (30%)	288 (35%)		0 (0%)		262 (32%)	26 (19%)	
CAD RAD 1/2	375 (39%)	375 (46%)		0 (0%)		340 (42%)	35 (25%)	
CAD RAD 3	135 (14%)	135 (17%)		0 (0%)		117 (14%)	18 (13%)	
CAD RAD 4A	97 (10%)	19 (2%)		78 (57%)		68 (8%)	29 (21%)	
CAD RAD 4B	61 (6%)	1 (0%)		60 (43%)		31 (4%)	30 (22%)	
CCTA stenosis >70% on any segment–N (%)	150 (16%)	20 (2%)		130 (94%)	<0.001	93 (11%)	57 (41%)	<0.001
CCTA stenosis >70% on any prox/mid/distal segment–N (%)	128 (13%)	0 (0%)		128 (93%)	<0.001	77 (9%)	51 (37%)	<0.001
CCTA stenosis >50% on any segment–N (%)	293 (31%)	155 (19%)		138 (100%)	<0.001	216 (26%)	77 (56%)	<0.001
CCTA stenosis >50% on any prox/mid/distal	269 (28%)	131 (16%)		138 (100%)	<0.001	196 (24%)	73 (53%)	<0.001
**CCTA burden of disease**								
Segment Involvement Score (SIS)—Mean (SD)	4.24 (4.16)	3.48 (3.82)		8.75 (3.13)	<0.001	3.88 (3.97)	6.38 (4.63)	<0.001
Calcified/Partially Calcified Plaque Segment Involvement Score–Mean (SD)	3.94 (4.06)	3.23 (3.74)		8.09 (3.41)	<0.001	3.60 (3.87)	5.91 (4.59)	<0.001
Calcified Plaque Segment Involvement Score–Mean (SD)	1.48 (2.65)	1.30 (2.46)		2.55 (3.36)	<0.001	1.36 (2.52)	2.17 (3.25)	<0.001
Partially Calcified Plaque Segment Involvement Score–Mean (SD)	2.46 (3.25)	1.94 (2.84)		5.54 (3.76)	<0.001	2.24 (3.04)	3.73 (4.07)	<0.001
Non-Calcified Plaque Segment Involvement Score–Mean (SD)	0.31 (0.78)	0.24 (0.66)		0.67 (1.22)	<0.001	0.28 (0.73)	0.48 (1.03)	0.005
**SPECT**								
Any ischemia—N (%)	138 (14%)	85 (10%)		53 (38%)	<0.001	0 (0%)	138 (100%)	<0.001
Significant ischemia (>10%)—N (%)	39 (4%)	14 (2%)		25 (18%)	<0.001	0 (0%)	39 (28%)	<0.001
Scar–N (%)	158 (17%)	102 (12%)		56 (41%)	<0.001	85 (10%)	73 (53%)	<0.001
LVEF–N (%)					0.034			<0.001
<35%	61 (6%)	49 (6%)		12 (9%)		46 (6%)	15 (11%)	
35–50%	96 (10%)	75 (9%)		21 (15%)		64 (8%)	32 (23%)	
>50%	799 (84%)	694 (85%)		105 (76%)		708 (87%)	91 (66%)	

**Table 3:** Clinical outcomes of the patients included in this study.

		Total N = 956	CCTA obstructive stenosis			SPECT—ischemia		
			No	Yes	*P value*	No	Yes	*P value*
			N = 818	N = 138		N = 818	N = 138	
MACE	Incidence rate (per 1000 person-year)	29.2	22.9	69	<0.001	22.9	69	<0.001
	N (%)	102 (11%)	69 (8%)	33 (24%)	<0.001	69 (8%)	33 (24%)	<0.001
All-cause death–N (%)		55 (6%)	41 (5%)	14 (10%)	0.017	38 (5%)	17 (12%)	<0.001
Myocardial infarction–N (%)		29 (3%)	19 (2%)	10 (7%)	0.002	19 (2%)	10 (7%)	0.002
PCI 90-days post imaging–N (%)		19 (2%)	9 (1%)	10 (7%)	<0.001	12 (1%)	7 (5%)	0.005
CABG 90-days post imaging–N (%)		8 (1%)	2 (0%)	6 (4%)	<0.001	4 (0%)	4 (3%)	0.004

### Feature selection

**Fig 1:**
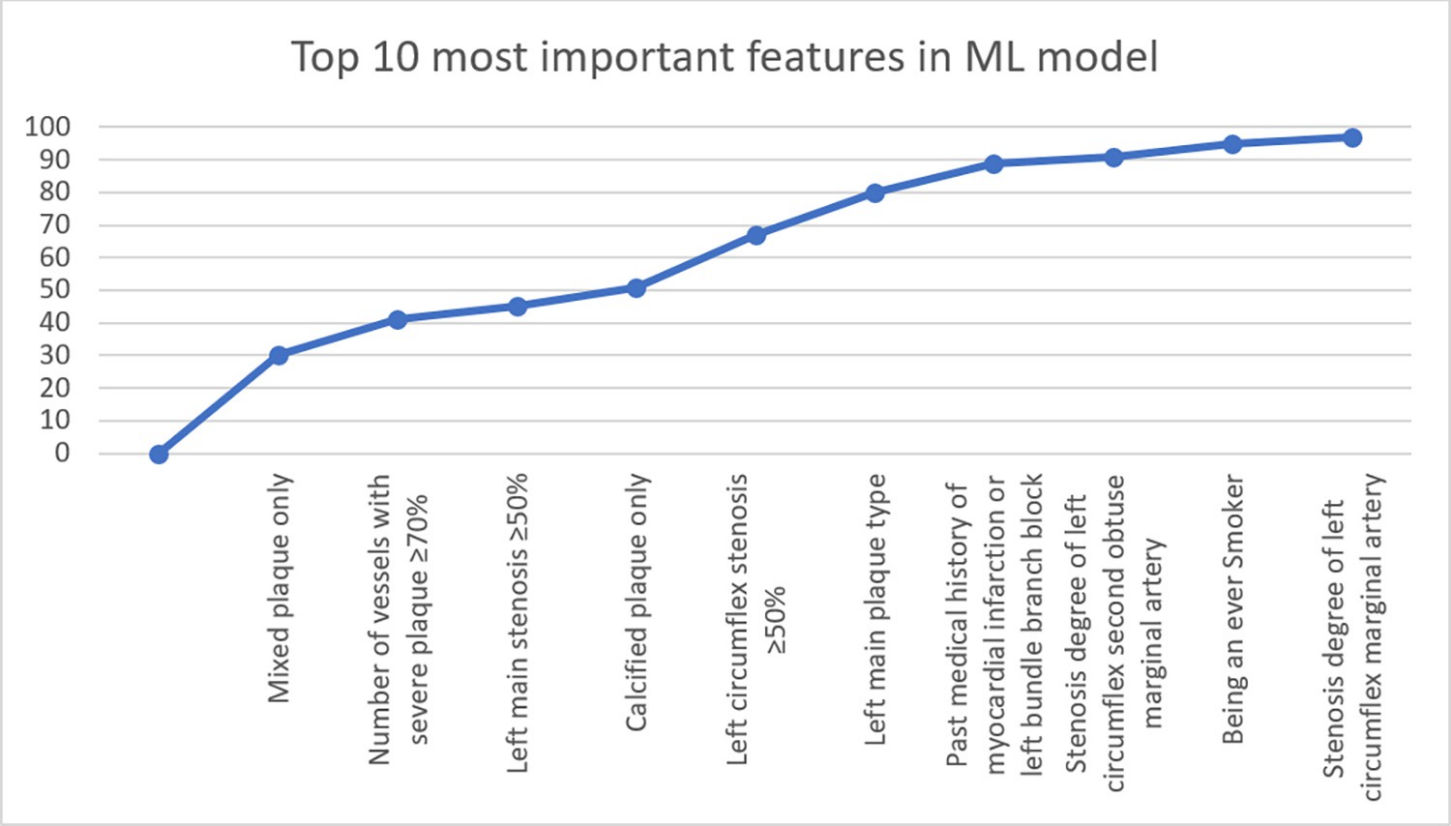
Feature importance ranking of the machine learning model for predictive MACE. Only the top 10 most important features were labelled.

The ANOVA-F value is calculated for each feature, and the features are ranked by their F-values in descending order. Fig 1 shows the top ten predictive features identified by ANOVA-F test. These features were used in building the ML model.

### AutoML model of MACE

During a median of 31 months of follow-up (with an interquartile range of 12 to 65 months), 102 patients (10.7%, or 29.2 events per 1000 person-years) experienced at least one major adverse cardiovascular event (MACE). Of these events, more than half (54%, or 55 patients) were all-cause mortality, while about a quarter (19 PCI and 8 CABG procedures) were due to the need for late revascularization. More detailed imaging and clinical evaluations were reported in our previous publication using this data [8].

The developed ML ensemble model demonstrated high specificity but moderate sensitivity in predicting major adverse cardiovascular events (MACE). The model had a sensitivity of 69.61% (95% confidence interval [CI] 59.71% to 78.33%), specificity of 99.77% (99.16% to 99.97%), and accuracy of 96.54%, as shown in Table 4.

**Table 4:** Accuracy measures of the machine learning model prediction of major adverse cardiovascular events.

Statistic	Value	95% CI
Sensitivity	69.61%	59.71% to 78.33%
Specificity	99.77%	99.16% to 99.97%
Positive Likelihood Ratio	297.23	74.01 to 1193.59
Negative Likelihood Ratio	0.3	0.23 to 0.41
MACE sample prevalence	0.11%	
Positive Predictive Value	24.10%	7.33% to 56.04%
Negative Predictive Value	99.97%	99.96% to 99.98%
Accuracy	96.54%	

### ML model interpretability

**Fig 2:**
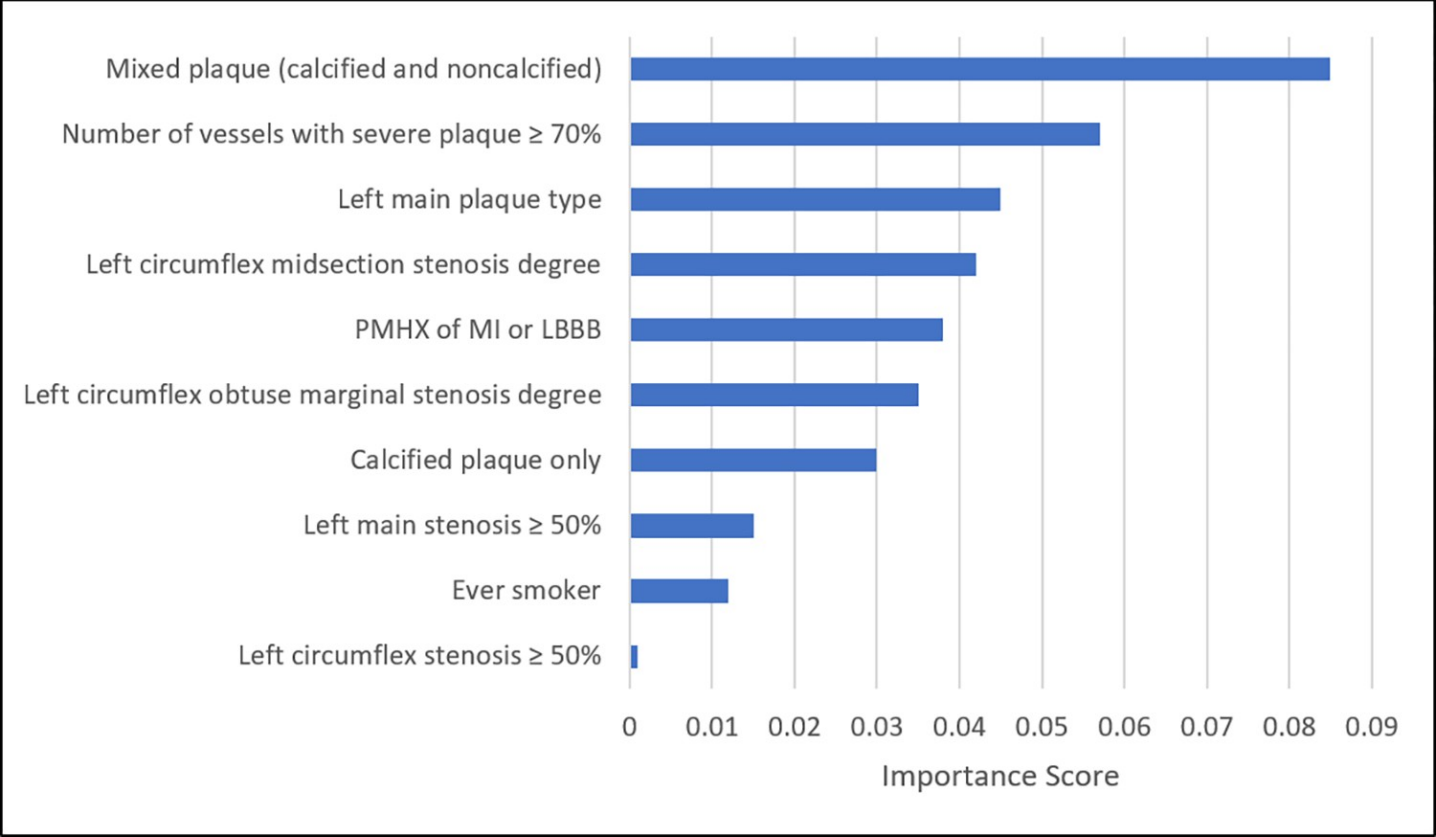
Permutation feature importance scores for the top ten features in the ensemble model for predicting MACE risk.

Global interpretability: Fig 2 shows the permutation feature importance scores for each feature in the ensemble model for predicting MACE risk. The most important 6 features were all CCTA parameters (segment involvement score, number of vessels with severe plaque ≥70, ≥50% stenosis in the left marginal coronary artery, calcified plaque, ≥50% stenosis in the left circumflex coronary artery, and plaque type in the left marginal coronary artery). The 7^th^ and 9^th^ most important features were clinical risk factors (past medical history of MI or left bundle branch block, followed by being an ever smoker), while the 8^th^ and 10^th^ features were also CCTA parameters (stenosis degree in the second obtuse marginal of the left circumflex artery, followed by stenosis category in the marginals of the left circumflex artery).

**Fig 3:**
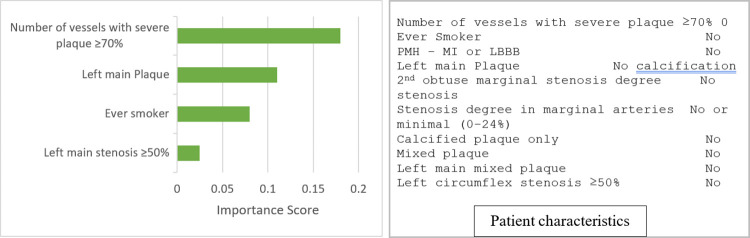
LIME explanation for a correctly predicted patient with a low risk of MACE.

**Fig 4:**
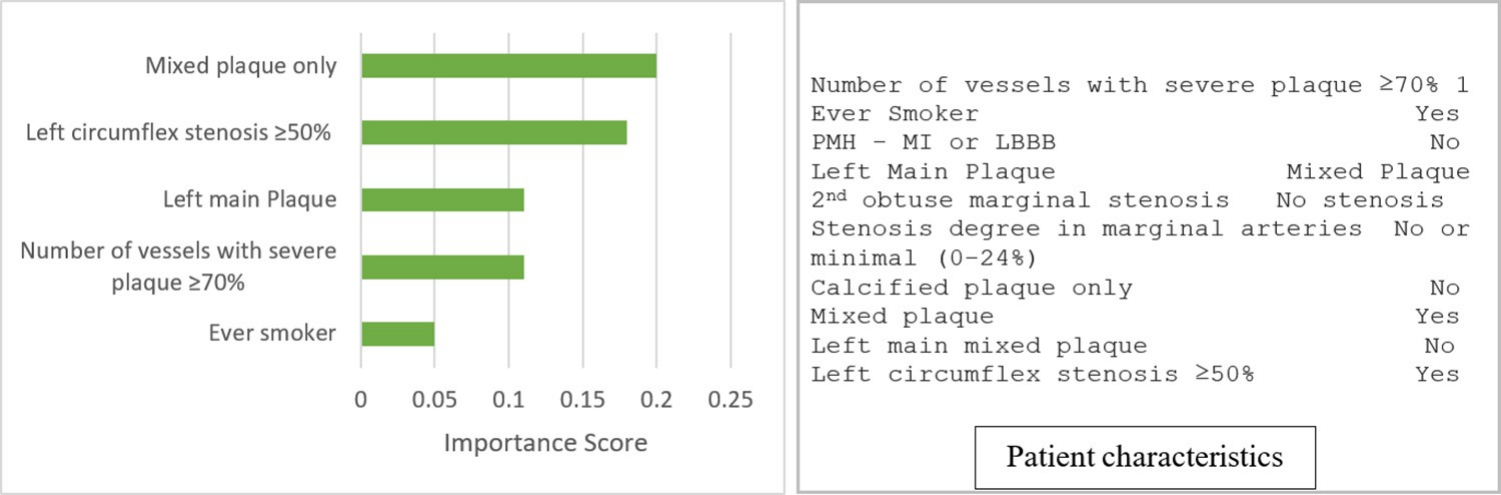
LIME explanation for a correctly predicted patient with a high risk of MACE.

Patient-level interpretability: Figs 3 and 4 show LIME explanations for two randomly selected patients. Fig 3 shows the explanation of a patient correctly predicted as low risk of MACE. The top four factors detected by the ensemble model that increased the risk of MACE were number of vessels with severe plaque ≥ 70%, left main plaque type, ever smoker, and left main stenosis ≥ 50%. Fig 4 shows the explanation of a patient correctly predicted as high risk of MACE. The top five features, including partially calcified plaque (calcified and noncalcified), Left circumflex stenosis ≥ 50%, Left main plaque type, number of vessels with severe plaque ≥ 70%, and ever-smoker contributed to the prediction.

Top ten global predictive features were identified in the study, eight of which were computed tomography angiography (CCTA) attributes related to plaque burden and stenosis, and two were clinical features. The most significant factor in predicting major adverse cardiovascular events (MACE) was the presence of partially calcified plaque (consisting of both calcified and noncalcified components). The number of vessels with severe plaque (greater than or equal to 70%) and a left main stenosis of greater than or equal to 50% were also found to be important predictors of MACE. Fig 1 illustrates the top ten predictive features and their incremental contribution to the predictive performance of the ML model. It is worth noting that features beyond the top ten had a minimal impact on the model’s performance.

## Discussion

We created an accurate, personalized ML method for predicting MACE risk for patients who undergo combined CCTA and SPECT imaging. This method combines all the available clinical, CCTA, and SPECT data variables, without making assumptions about the individual factors or their interplay. To optimize trust and gain a better understanding of how the ML predictions were made, we used a model-agnostic explanation of MACE predictions at the global and individual patient levels. Interestingly, out of the top 10 most contributory features to the ML prediction, most were CCTA attributes (8/10) and clinical variables (2/10). SPECT imaging features were not among the most important ones, as the first SPECT feature came in 19th place of all the 138 variables combined in global feature importance permutation. Notably, as seen in Fig 1, additional features contributed very little to the ML model predictive performance beyond the top 10 features.

Recent advancement in computed tomography assessment of the coronary vasculature has expanded the number of useful parameters that can be measured and collated. With the continued development, the amount, intricacy, and quality of the data arising from CCTA is increasing exponentially [35]. Evolving evidence also points to the utility of quantification of plaque burden for risk stratification of patients, with and without known CAD, for adverse events [36,37]. CCTA provides a comprehensive evaluation of coronary circulation by combining information on anatomy, function, and biology, which can be used to identify the risk level of patients and guide the selection of preventative measures that can be tailored to the individual [5,38–40]. AI and ML techniques have been developed and deployed to increase the efficiency and reliability of CCTA imaging by image acquirement, image processing, and automating quantification of plaque, stenosis, and inflammation [41–43]. Risk prediction was also attempted with promising results by applying similar ML algorithms to CCTA-derived perivascular fat attenuation and more complex plaque quantifications [44–46]. Furthermore, Slomka et al. reported a large multicenter study that tested a ML that relies on clinical and CCTA data to predict the 5-year all-cause death risk in patients suspected of CAD [47]. The ML model they evaluated achieved a higher area-under-the-curve when compared to risk assessment relying on CCTA severity scores alone. Similarly, Dey et al. evaluated the performance of a ML approach to predict cardiac death and myocardial infarction by integrating clinical and CCTA imaging features [48]. The model they derived achieved improved risk assessment when compared to the atherosclerotic cardiovascular disease risk score and the coronary artery calcium score. In a subsequent report, Dey et al. analyzed the revascularization prediction ability of a ML-ischemia risk score designed to identify hemodynamically significant CAD using CCTA. Their ML-derived risk score was able to predict future revascularizations better than a traditional risk model based on clinical data and stenosis.

Nuclear cardiology also attracted much attention from bioinformaticians and computer scientists due to the growing needs for such tools to keep up with the accelerating developments in the imaging technologies of the field. Particularly with a very widely available and frequently used non-invasive cardiac imaging modality such as SPECT, as well as its use as an adjunct diagnostic and prognostic assessment, ML tools have been developed to enhance its utility and predictive abilities [49]. ML techniques have improved the quality of the acquired SPECT image, enhanced quantification of various parameters, and booted its diagnostic and prognostic utilities for cardiac patients. One of the largest efforts in this domain is the REFINE SPECT registry of Slomka et al. which has produced several important reports [50]. One such report showed that a ML approach outperformed automatically-reported total perfusion defects as well as clinicians’ assessments in predicting early revascularization in 1980 patients suspected of CAD [51]. That same report also offered explainability by ranking the most important SPECT features. In subsequent iterations, they reported their work in finding the minimum number of variables that would help retain the ability of their ML model in predicting MACE incidents [52] and in predicting abnormal MPI from pre-test information [53]. Our work builds on the previous efforts and takes it one step further in using ML techniques to integrate a large number of variables emerging from CCTA imaging, SPECT imaging, as well as clinical patient data. This approach is more real-world-like as CCTA and SPECT images are available for a large proportion of patients suspected of, or already diagnosed with, CAD. The ML model we evaluated in this study allows seamless integration of these parameters and offers explanations at the global as well as the individual patient levels. Interestingly, CCTA parameters showed higher predictive raking in how they contribute to the overall performance of the ML model in predicting MACE incidents. Of the top ten most important features in the model, 8 came from CCTA imaging and 2 from the clinical data, while no SPECT features were among the top 10 list.

Artificial intelligence has been applied in various ways in cardiovascular medicine, including using ML techniques for diagnostic procedures involving imaging techniques and biomarkers, as well as using predictive analytics for personalized therapies with the goal of improving outcomes [54]. ML algorithms have also been used to predict the risk of cardiovascular diseases [55–57], in cardiovascular imaging [54,58–60], to forecast outcomes following revascularization procedures [61,62], and to identify potential new drug targets [63–65]. Using ML in clinical decision making can provide a more comprehensive analysis of all available data on patients suspected of CAD, which may be difficult for physicians to do objectively [66–69]. Cardiologists have to integrate several data sources, including clinical data, CT imaging, and SPECT stress testing, among others, to make clinical decisions, but there is no consistent method for integrating all of this information [70–72]. Additionally, guidelines recommend including certain variables in the myocardial perfusion imaging (MPI) report, but there is currently no standardized way to include them in the report [73]. The interpretation of myocardial perfusion imaging (MPI) is subjective and the risk assessment of a patient based on clinical, stress test, and imaging results can vary depending on the physician’s knowledge and experience, as well as the difficulty of properly evaluating individual factors. It is unlikely that physicians would be able to accurately and consistently consider all clinical and imaging factors for risk assessment in an individual patient scenario, whether they are interpreting the imaging report or treating the patient. Using explainable ML models offers a more reliable computational method for integrating all available information, while providing an explanation for the prediction at both the global and individual patient levels. The potential benefit of this ML predictive model also extends to improving accuracy and consistency not only across medical centers, including those with different amount of experience, medical care level, and across varying geographical locations and socioeconomic disparities, but also within such centers among their clinical and imaging physicians [74]. Furthermore, the intention behind the two-level explainability reporting is to help contribute to ML model transparency, interpretability, and trustworthiness. Since the “black box” nature of most ML models is one of the challenges of its understanding and adoption, this would help to alleviate the tendency to mistrust the unknown behavior of ML models.

### Limitations

The sample size in this single-center study was relatively small for a ML study, and follow-up was limited to a median of 31 months, however, the results were significant. Furthermore, additional research is needed to determine the utility for prospective clinical implementation and to validate the score in multi-center and external studies. Additional variables could be considered in future studies, including those from other cardiovascular imaging modalities, and it may be useful to evaluate the ML risk stratification in specific subpopulations, such as patients with suspected disease or early revascularization. It is also unknown how well the score will extrapolate to different centers, patient populations, and follow-up times. Other ML approaches may provide more advanced risk prediction but would likely require larger datasets and more sophisticated computational resources. Finally, because we included patients that had undergone both CCTA and SPECT imaging, the study cohort may have included higher risk patients, which may partially explain the lower sensitivity. However, this was mitigated by excluding patients with known CAD.

## Conclusion

We developed an ML model that can accurately predict risk of developing a MACE in patients suspected of CAD undergoing SPECT MPI and CCTA. ML feature-ranking can also show, at a global- as well as a patient-level, which features are key in making such a prediction. Of the top 10 most important predictive features, 8 features came from CCTA imaging and 2 came from clinical variables. ML explainable models of MACE prediction using clinical and imaging variables can help ensure high accuracy of risk prediction as well as consistency within and across healthcare centers and systems.

## Supporting information

S1 File Supplementary Tables: S1 Table Feature importance scores as calculated by Functional ANOVA; S2 Table Complete list of variables and parameters included in the machine learning model. (DOCX)
