# Supplementary material for: Patient-level explainable machine learning to predict major adverse cardiovascular events from SPECT MPI and CCTA imaging
Source: PLoS One. 2023 Nov 15;18(11):e0291451. doi: 10.1371/journal.pone.0291451 (PMC10651041; doi:10.1371/journal.pone.0291451)
Supplement: S1 File — (DOCX) [file pone.0291451.s001.docx]

Supplementary Tables: Table S1: Feature importance scores as calculated by Functional ANOVA. (Full list of abbreviations can be found in Suppl-Table S2)

Table S2: Complete list of variables and parameters included in the machine learning model.

| # | Source | Variable name |
| --- | --- | --- |
| 1 | Clinical variables | Age |
| 2 |  | Race |
| 3 |  | Gender |
| 4 |  | Height |
| 5 |  | Weight |
| 6 |  | Body Mass Index (BMI) |
| 7 |  | Hypertension |
| 8 |  | Diabetes |
| 9 |  | Dyslipidemia |
| 10 |  | Ever Smoker |
| 11 |  | Heart Failure |
| 12 |  | PMH - Cancer |
| 13 |  | PMH – Cerebrovascular Accident (CVA) |
| 14 |  | PMH - Pulmonary Embolus (PE) |
| 15 |  | PMH – Peripheral Arterial Disease (PAD) |
| 16 |  | PMH - PAD or PE |
| 17 |  | PMH – Myocardial Infarction (MI) |
| 18 |  | Past Medical History - Left Bundle Branch Block |
| 19 |  | Past Medical History - MI or LBBB |
| 20 |  | Family History – Congestive Heart Failure (CHF) |
| 21 |  | Asymptomatic |
| 22 |  | Shortness of Breath |
| 23 |  | Syncope |
| 24 |  | Chest Pain or Shortness of Breath |
| 25 |  | Creatinine |
| 26 |  | Estimated Glomerular Filtration Rate (GFR) |
| 27 |  | Estimated GFR/10 |
| 28 |  | Total Cholesterol |
| 29 |  | High Density Lipoprotein (HDL) |
| 30 |  | Low Density Lipoprotein (LDL) |
| 31 |  | Triglycerides |
| 32 |  | HbA1C |
| 33 |  | High Sensitivity CRP |
| 34 |  | Aspirin |
| 35 |  | Clopidogrel |
| 36 |  | Aspirin/Clopidogrel |
| 37 |  | Statin |
| 38 |  | Angiotensin Converting Enzyme (ACE) Inhibitors |
| 39 |  | Angiotensin Receptor Blockers |
| 40 |  | ACE/ARB |
| 41 |  | Beta Blockers |
| 42 |  | Calcium Channel Blockers |
|  |  |  |
| 1 | CCTA variables | Coronary Artery Calcium Score (CACS) |
| 2 |  | Left Main (LM) plaque type |
| 3 |  | LM stenosis degree |
| 4 |  | Left Arterial Descending (LAD) plaque type |
| 5 |  | LAD proximal (p) stenosis degree |
| 6 |  | LAD (mid) m plaque type |
| 7 |  | LADm stenosis degree |
| 8 |  | LAD distal (d) plaque type |
| 9 |  | LADd stenosis degree |
| 10 |  | LADd1 plaque type |
| 11 |  | LADd1 stenosis degree |
| 12 |  | LADd2 plaque type |
| 13 |  | LADd2 stenosis degree |
| 14 |  | Left Circumflex (LCXp) plaque type |
| 15 |  | LCXp stenosis degree |
| 16 |  | LCXm plaque type |
| 17 |  | LCXm stenosis degree |
| 18 |  | LCXd plaque type |
| 19 |  | LCXd stenosis degree |
| 20 |  | LCX obtuse marginal (om)1 plaque type |
| 21 |  | LCXom1 stenosis degree |
| 22 |  | LCXom2 plaque type |
| 23 |  | LCXom2 stenosis degree |
| 24 |  | LCXpd plaque type |
| 25 |  | LCXpd stenosis degree |
| 26 |  | Right Coronary Artery (RCA)p plaque type |
| 27 |  | RCAp stenosis degree |
| 28 |  | RCAm plaque type |
| 29 |  | RCAm stenosis degree |
| 30 |  | RCAd plaque type |
| 31 |  | RCAd stenosis degree |
| 32 |  | RCApd plaque type |
| 33 |  | RCApd stenosis degree |
| 34 |  | RCApl plaque type |
| 35 |  | RCApl stenosis degree |
| 36 |  | Ramus plaque type |
| 37 |  | Ramus stenosis degree |
| 38 |  | LM stenosis category |
| 39 |  | LADp stenosis category |
| 40 |  | LADm stenosis category |
| 41 |  | LADd stenosis category |
| 42 |  | LADd1 stenosis category |
| 43 |  | LADd2 stenosis category |
| 44 |  | LCXp stenosis category |
| 45 |  | LCXm stenosis category |
| 46 |  | LCXd stenosis category |
| 47 |  | LCXom1 stenosis category |
| 48 |  | LCXom2 stenosis category |
| 49 |  | LCXpd stenosis category |
| 50 |  | RCAp stenosis category |
| 51 |  | RCAm stenosis category |
| 52 |  | RCAd stenosis category |
| 53 |  | RCApd stenosis category |
| 54 |  | RCApl stenosis category |
| 55 |  | Ramus stenosis category |
| 56 |  | Calcified Plaque Segment Involvement Score |
| 57 |  | Mixed Plaque Segment Involvement Score |
| 58 |  | Non-Calcified Plaque Segment Involvement Score |
| 59 |  | Segment Involvement Score (SIS) |
| 60 |  | Calcified/Mixed Plaque Segment Involvement Score |
| 61 |  | Total Plaque Segment Involvement Score>3 |
| 62 |  | CCTA obstructive stenosis |
| 63 |  | CCTA obstructive stenosis on any segment |
| 64 |  | CCTA stenosis >70% on any segment |
| 65 |  | CCTA stenosis >70% on any prox/mid/distal segment |
| 66 |  | CCTA stenosis >50% on any segment |
| 67 |  | CCTA stenosis >50% on any prox/mid/distal segment |
| 68 |  | CCTA Modified Duke Score |
| 69 |  | CCTA CAD-RAD |
| 70 |  | CCTA degree of stenosis |
| 71 |  | CCTA Non-obstructive stenosis |
| 72 |  | Calcified plaque only |
| 73 |  | Non-calcified plaque only |
| 74 |  | Mixed plaque only |
| 75 |  | Segment Stenosis Score |
| 76 |  | Number of vessels with plaque<50% |
| 77 |  | Number of vessels with moderate plaque (50-69%) |
| 78 |  | Number of vessels with severe plaque ≥70% |
| 79 |  | Number of vessels with moderate or above plaque ≥50% |
| 80 |  | LM stenosis≥50% |
| 81 |  | Left Anterior Descending (LAD) stenosis≥50% |
| 82 |  | LCX stenosis≥50% |
| 83 |  | RCA stenosis≥50% |
|  |  |  |
| 1 | SPECT variables | SPECT % Ischemia |
| 2 |  | SPECT Scar |
| 3 |  | SPECT Total Perfusion Burden |
| 4 |  | SPECT End Diastolic Volume |
| 5 |  | SPECT End Systolic Volume |
| 6 |  | SPECT Left-Ventricular Ejection Fraction |
| 7 |  | Any ischemia by SPECT |
| 8 |  | SPECT Significant Ischemia (>10%) |
| 9 |  | Any scar by SPECT |
| 10 |  | SPECT Significant Perfusion Burden (>10%) |
| 11 |  | SPECT LVEF <40% |
| 12 |  | SPECT LVEF <55% |
